# Supplementary material for: Cyclin D1—Cdk4 regulates neuronal activity through phosphorylation of GABAA receptors
Source: Cell Mol Life Sci. 2023 Sep 8;80(10):280. doi: 10.1007/s00018-023-04920-7 (PMC10491536; doi:10.1007/s00018-023-04920-7)
Supplement: Supplementary file 1 — Supplementary file1 (DOCX 241 KB) [file 18_2023_4920_MOESM1_ESM.docx]

**SUPPLEMENTARY DATA**

| **Accession** | **Description** | **Score** | **Coverage** | **# Proteins** | **# Unique Peptides** | **# Peptides** | **MW (kDa)** |
| --- | --- | --- | --- | --- | --- | --- | --- |
| Q9D6F4 | GBRA4_MOUSE | 309,37 | 33,70 | 1 | 16 | 17 | 60,8 |
|  | | | | | | | |
| **Sample** | **Peptide sequence** | **Modifications** | **phoSite Probab** | **q-Value** | **MH+ (Da)** | **ΔM (ppm)** | **RT (min)** |
| **ATP** | TSAVQESSEATPK |  |  | 0,002 | 1334,64 | -1,09 | 15,99 |
|  | TSAVQESSEA**t**PK | T11(Phospho) | T(11): 100,0 | 0,012 | 1414,61 | -1,91 | 15,92 |
|  | AHLASSPNPFSR |  |  | 0,038 | 1283,64 | -3,28 | 19,81 |
|  | AHLAS**s**PNPFSR | S6(Phospho) | S(6): 98,3 | 0,131 | 1363,61 | -5,17 | 20,50 |
| **no ATP** | TSAVQESSEATPK |  |  | 0,0000 | 1334,64 | -0,91 | 16,33 |
|  | AHLASSPNPFSR |  |  | 0,0000 | 1283,64 | -3,57 | 20,01 |

***Supplementary Table 1.*** ***The α4 subunit of GABA_A_ receptors is phosphorylated at T423 and S431 by Ccnd1–Cdk4.*** *The GST-Ct-α4 wild type was used in an in vitro kinase assay with Ccnd1–Cdk4, in the presence or absence of ATP (no ATP), and the Chymotryptic peptide molecular masses were analyzed by liquid chromatography-mass spectrometry. MH+: proton adduct;* *ΔM: mass difference; RT: retention time; q-value: adjusted p-value*. *Both T423 and S431 of α4 subunit (highlighted in red) were found to be phosphorylated by Ccnd1–Cdk4.*

***Supplementary Figure 1.*** ***Ccnd1 interacts with the α4 subunit of GABA_A_ receptors.*** *HEK-293T cells were transfected with the C terminus of α4 subunit (354-552aa, tagged with HA: HA-Ct-α4) and Ccnd1 (tagged with Flag: Flag-Ccnd1). At 24 hours after transfection, Flag-Ccnd1 was precipitated using αFlag agarose beads (Sigma). Input (IN) and immunoprecipitation (IP) samples were analyzed by western blot using anti-HA and anti-Flag antibodies, to detect co-IP of the C terminus of α4 with Ccnd1.*


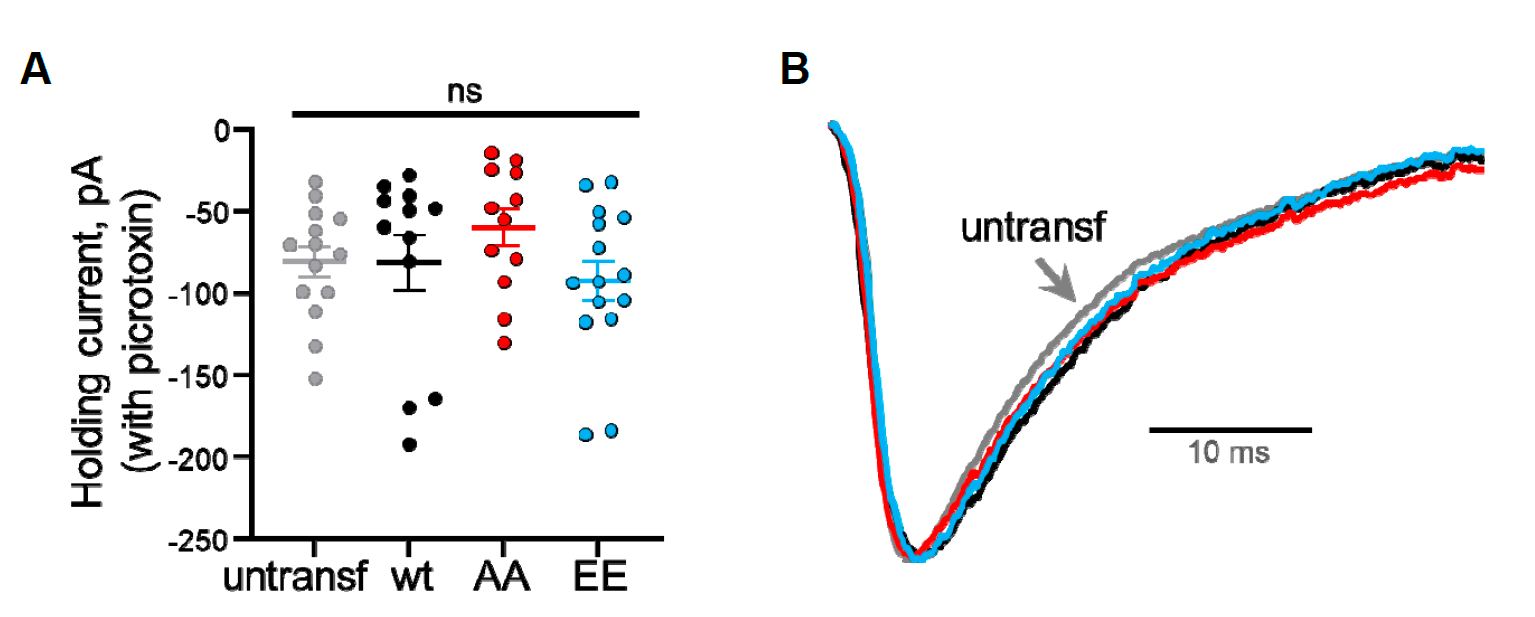


***Supplementary Figure 2. Expression of recombinant α4 in culture hippocampal slices does not change non-GABA_A_ receptor currents and alters mIPSC kinetics.*** *A) Quantification of absolute values for holding current after picrotoxin addition, from neurons plotted in Fig. 6, A and B. B) Scaled average mIPSCs from neurons plotted in Fig. 6 C and D, to compare the kinetics of the responses for each condition. The average decay times of mIPSCs were: 11.71±0.08 ms (untransfected neurons), 13.73±0.07 ms (wt), 13.04±0.08 ms (AA), 12.86±0.08 ms (EE). For all panels, wild-type α4 is represented with black symbols, T423A/S431A (AA) α4 with red symbols, T423E/S431E (EE) α4 with blue symbols and untransfected neurons with grey symbols.*
